# Supplementary material for: Application of polymersomes engineered to target p32 protein for detection of small breast tumors in mice
Source: Oncotarget. 2018 Apr 10;9(27):18682–97. doi: 10.18632/oncotarget.24588 (PMC5922347; doi:10.18632/oncotarget.24588)
Supplement: Supplementary file 1 [file oncotarget-09-18682-s001.pdf]

Application of polymersomes engineered to target p32 protein for detection of small breast tumors in mice

SUPPLEMENTARY MATERIALS

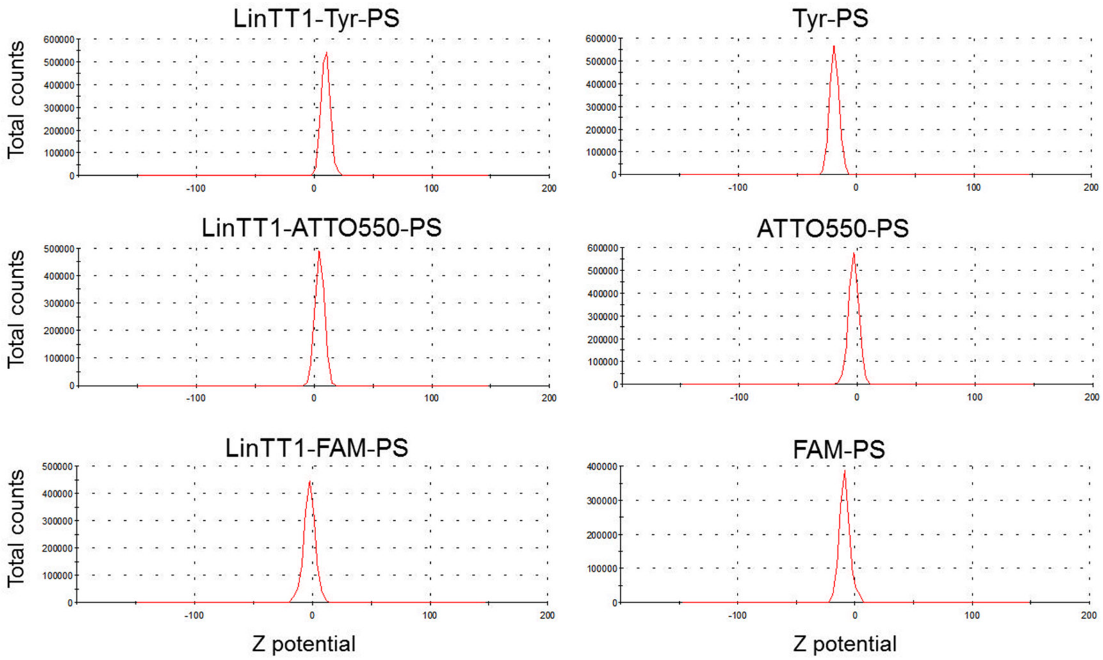

Supplementary Figure 1: Z potential of polymersomes. Z potential of the different polymersomes measured with the Zetasizer Nano ZS, Malvern Instruments.

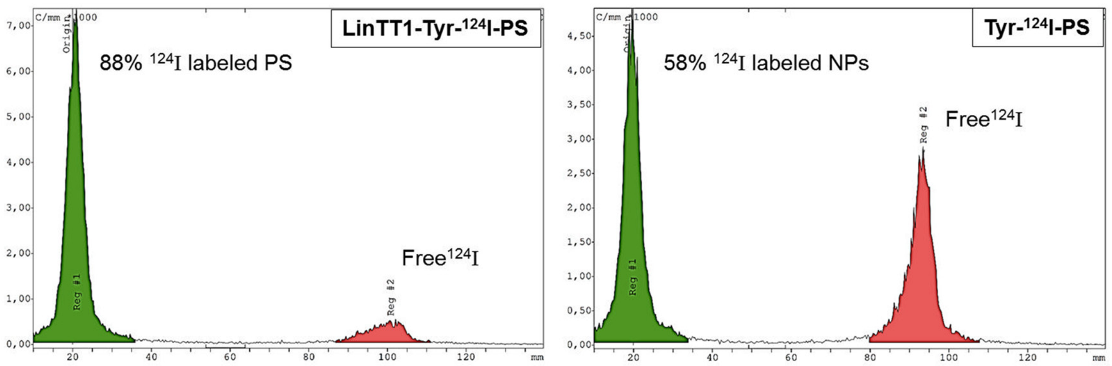

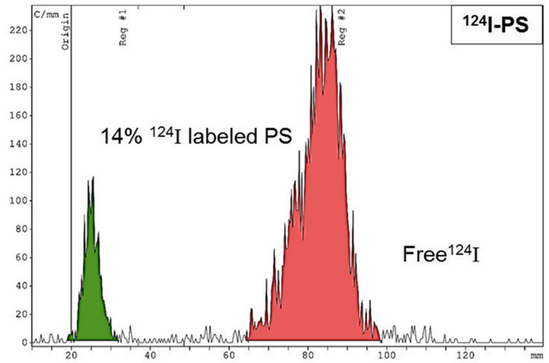

Compound	Yield of radiolabeling (%)
LinTT1-Tyr-^{124}I-PS	48 ± 9
Tyr-^{124}I-PS	43 ± 2

Supplementary Figure 2: Characterization of radiolabeled polymersomes. Chromatogram obtained by TLC before the purification of radiolabeled polymersomes showing the percentage of ^{124}I labeled polymersomes (green) and free ^{124}I (red). Table of the radiolabeling yield measured after purification.

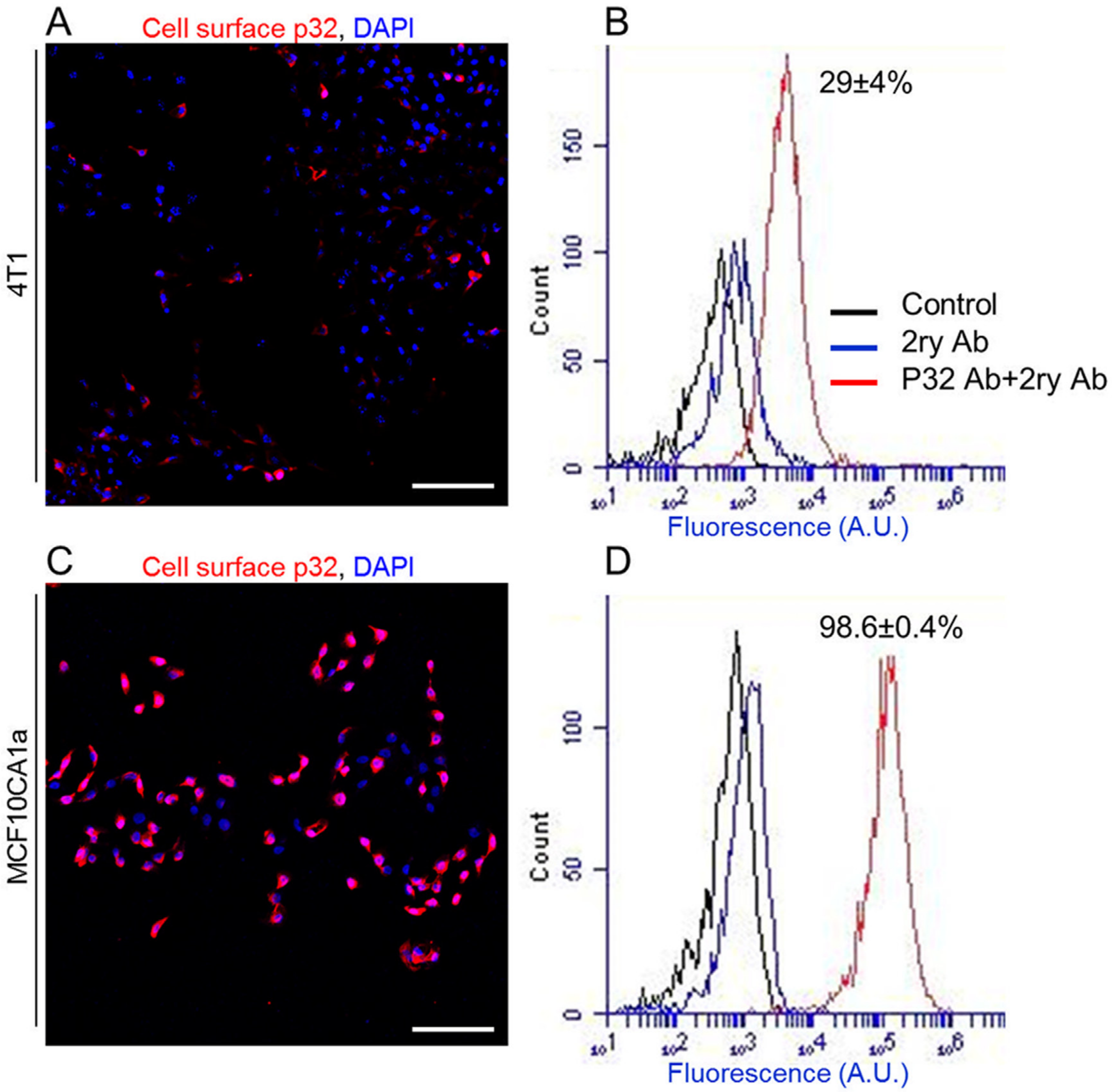

Supplementary Figure 3: Expression of p32 on cell surface. **(A** and **C)** Fluorescence confocal microscopy images of 4T1 (A) and MCF-10CA1a cells (C) immunostained for cell surface p32 (red) and couterstained with DAPI (blue). Scale=100 μm. **(B** and **D)** Flow cytometry for the quantification of cell surface p32 in 4T1 (B) and MCF-10CA1a cells (D). Cells were incubated with anti-p32 antibody and Alexa647-conjugated secondary antibody (red line) or only with the Alexa647-conjugated secondary antibody (blue line). Black lines are the control cells without incubation. The graphic represents the % of fluorescent cells (count). N=3.

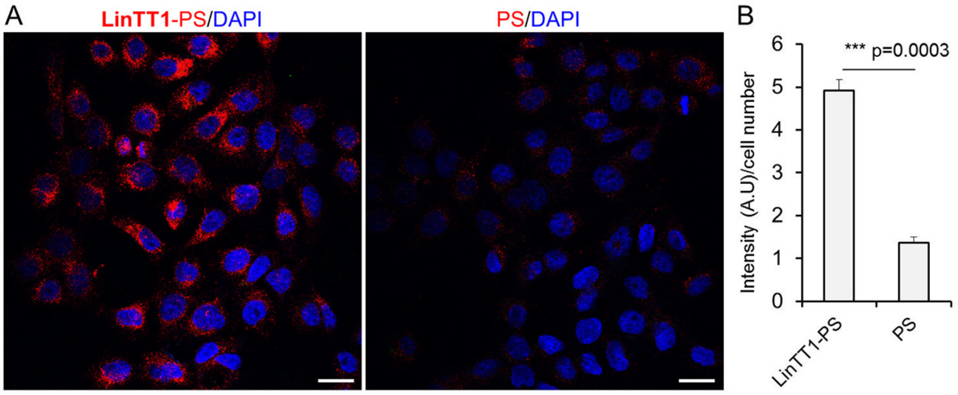

Supplementary Figure 4: Binding of LinTT1-PS to cultured MCF10CA1a breast tumor cells. **(A)** Fluorescence confocal microscopy images of MCF10CA1a cells incubated with LinTT1-PS or non-targeted Rho-PS for 1 h. The polymersomes were labeled with Rhodamine B octadecyl ester at 5% of molarity (red) and the nuclei were counterstained with DAPI (blue). Scale bar=20μm. **(B)** Quantification of the polymersome cell uptake using Image J software. N=3. Error bars=+SEM. For the polymersome labeling with Rhodamine B, Rodamine B octadecyl ester was mixed at 5% of molarity with the co-polymer in 0.5mL of acetone and the solvent was evaporated with nitrogen flow to form the polymer film. The polymersomes were formed and purified as previously described in materials and method section. 4T1 cells ($7x10^5$) were seeded on glass coverslips in a 24-well plate and the next day incubated with Rhodamine B-labeled polymersomes (0.5mg polymer/mL) at 37°C for 1 h. Cells were washed with PBS, fixed with 4% paraformaldehyde and counterstained with 1μg/mL of DAPI. Cells were examined by fluorescence confocal microscopy using the Zeiss LSM710 instrument.

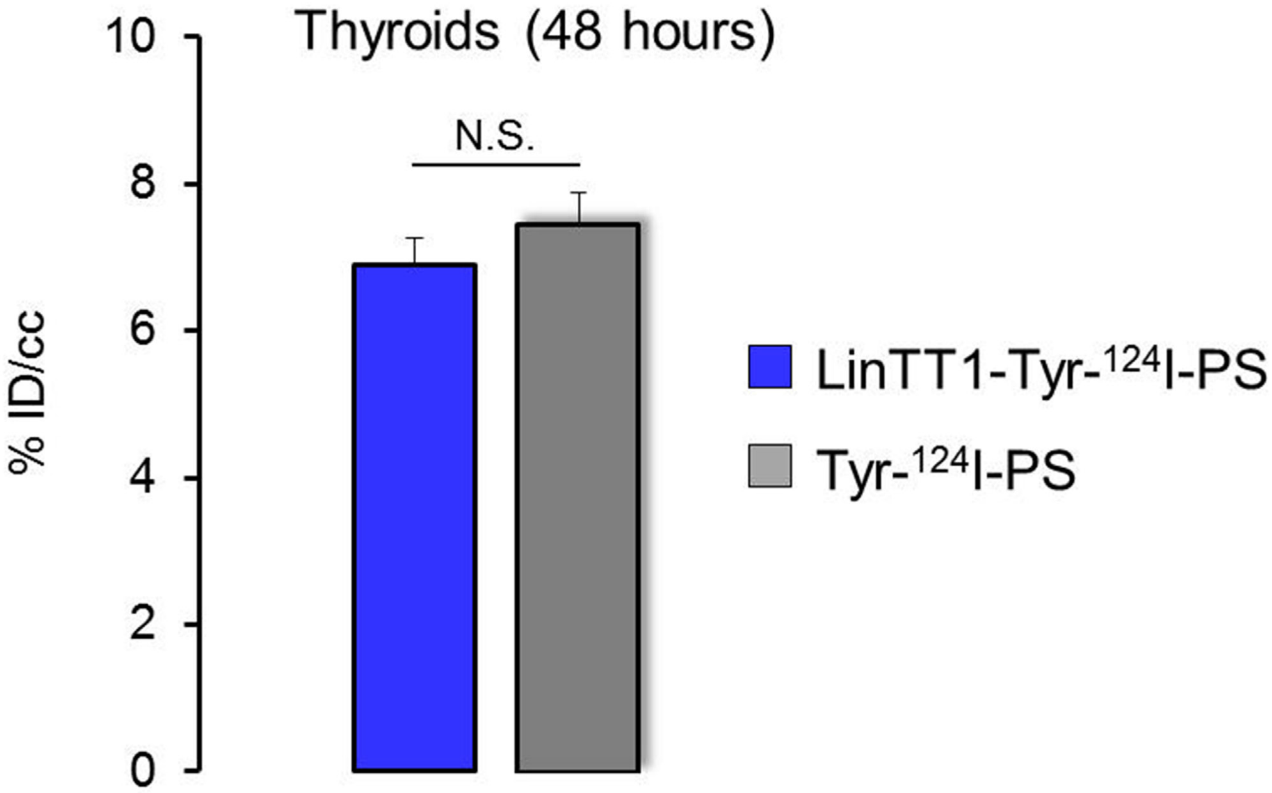

Supplementary Figure 5: Accumulation of radiolabeled polymersomes in thyroids. Percentage of injected dose per cubic centimeter (ID/cc) in thyroids after 48 hours of polymersome injection. The signal was quantified from the PET images. N=5 mice. Error bar=+SEM.

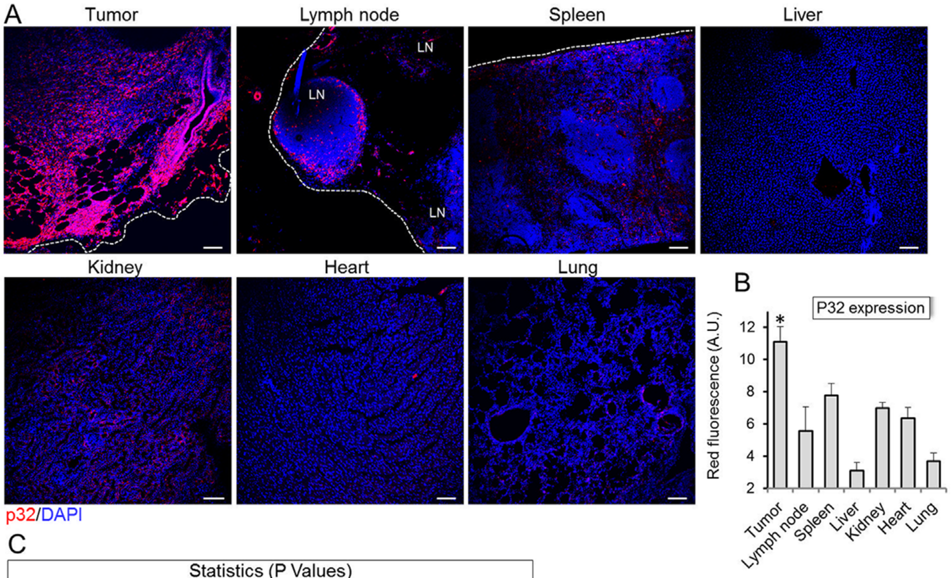

C

Statistics (P Values)

Tumor		0.0003	0.0131	0.0000	0.0035	0.0012	0.0000
Lymph	0.0003		0.0849	0.0525	0.2544	0.5196	0.1321
Spleen	0.0131	0.0849		0.0014	0.5166	0.2525	0.0039
Liver	0.0000	0.0525	0.0014		0.0052	0.0148	0.6117
Kidney	0.0035	0.2544	0.5166	0.0052		0.6057	0.0145
Heart	0.0012	0.5196	0.2525	0.0148	0.6057		0.0403
Lung	0.0000	0.1321	0.0039	0.6117	0.0145	0.0403	

Supplementary Figure 6: P32 expression in 4T1 tumor and organs. (A) Confocal microscope pictures from 4T1 tumor and organs sections. Tumor and organs were excised after 24 hours of LinTT1-FAM-PS i.v. injection into 4T1 bearing mice and sectioned and immunostained for FAM and p32 protein. Green: LinTT1-FAM-PS; red: p32; blue: DAPI. Scale bar=100µm. **(B)** Quantification of the p32 expression in tumor and organs with Image J software. N=3. Error bar=+SEM. **(C)** Table of the statistics of p32 expression. N=3.

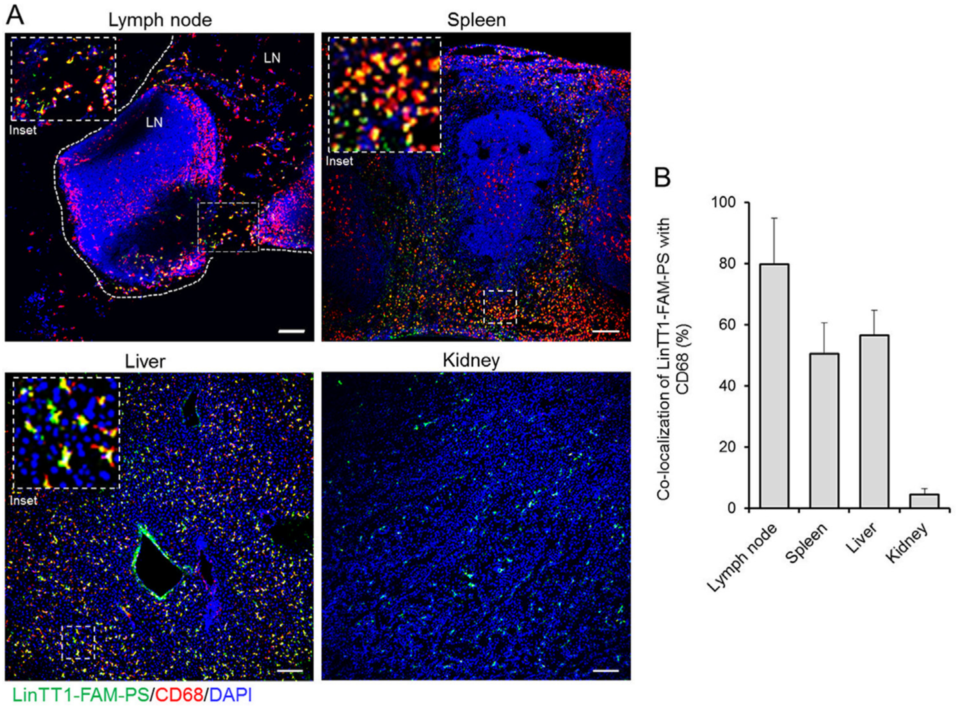

Supplementary Figure 7: Colocalization of LinTT1-PS with macrophage markers in organs. (A) Confocal pictures of organs sections immunostained for FAM and CD68 and counterstained with DAPI. Green: LinTT1-FAM-PS; red: CD68, CD11b, CD206; blue: DAPI counterstaining. Scale bar=100μm. LN=lymph node. **(B)** Quantification of the colocalization of LinTT1-FAM-PS and CD68 in organs with FLUOVIEW Viewer software. Error bar=+SEM.

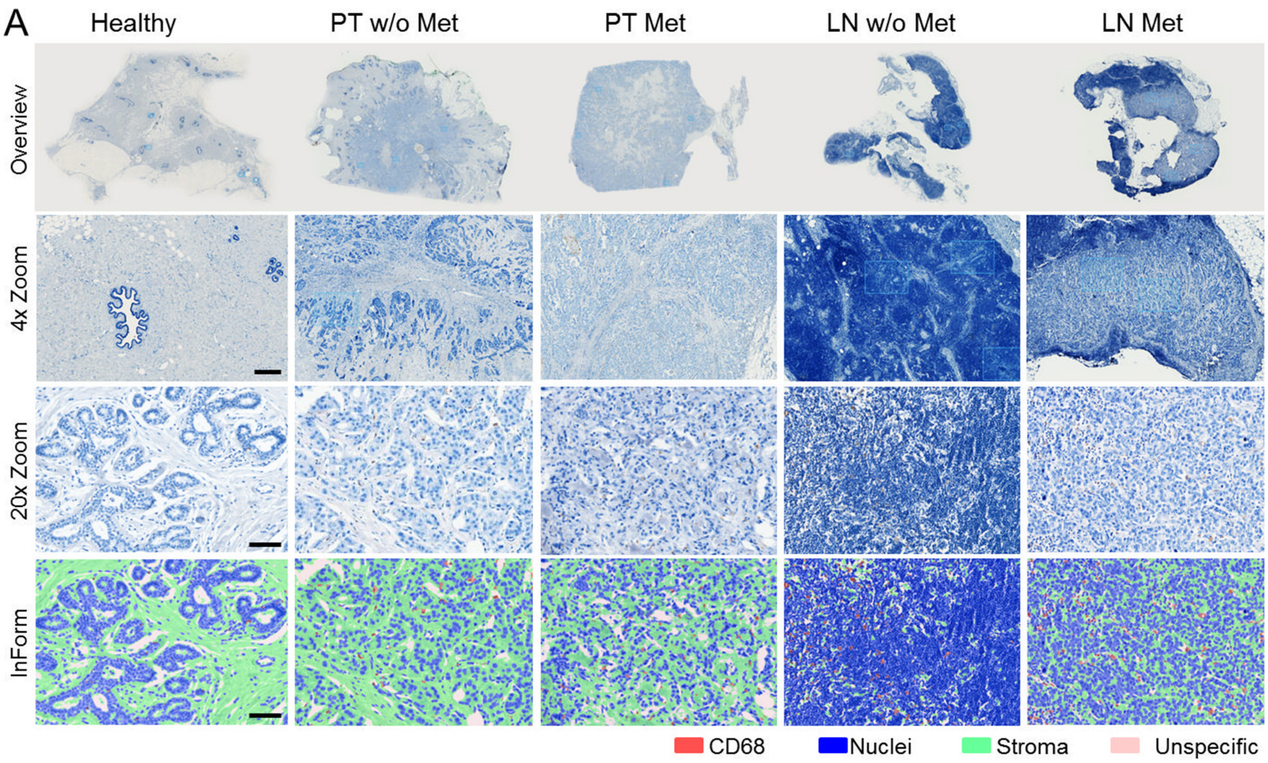

Supplementary Figure 8: CD68+ macrophages in human tissue samples. Immunohistochemistry CD68 (DAB) staining of FFPE human sections of healthy breast tissue (healthy), Primary Tumors with and without metastasis (PT Met and PT w/o Met, respectively) and their correspondent sentinel lymph nodes (LN Met and LN w/o Met, respectively), in N=10 patients/group. **(A)** Representative overview of the paraffin cut and 4x magnification evidence the healthy breast ultrastructure (lobuli and ducts surrounded by large amounts of fat tissue), as well as the presence of the primary tumors and lymph node metastasis surrounded by remaining healthy tissue.

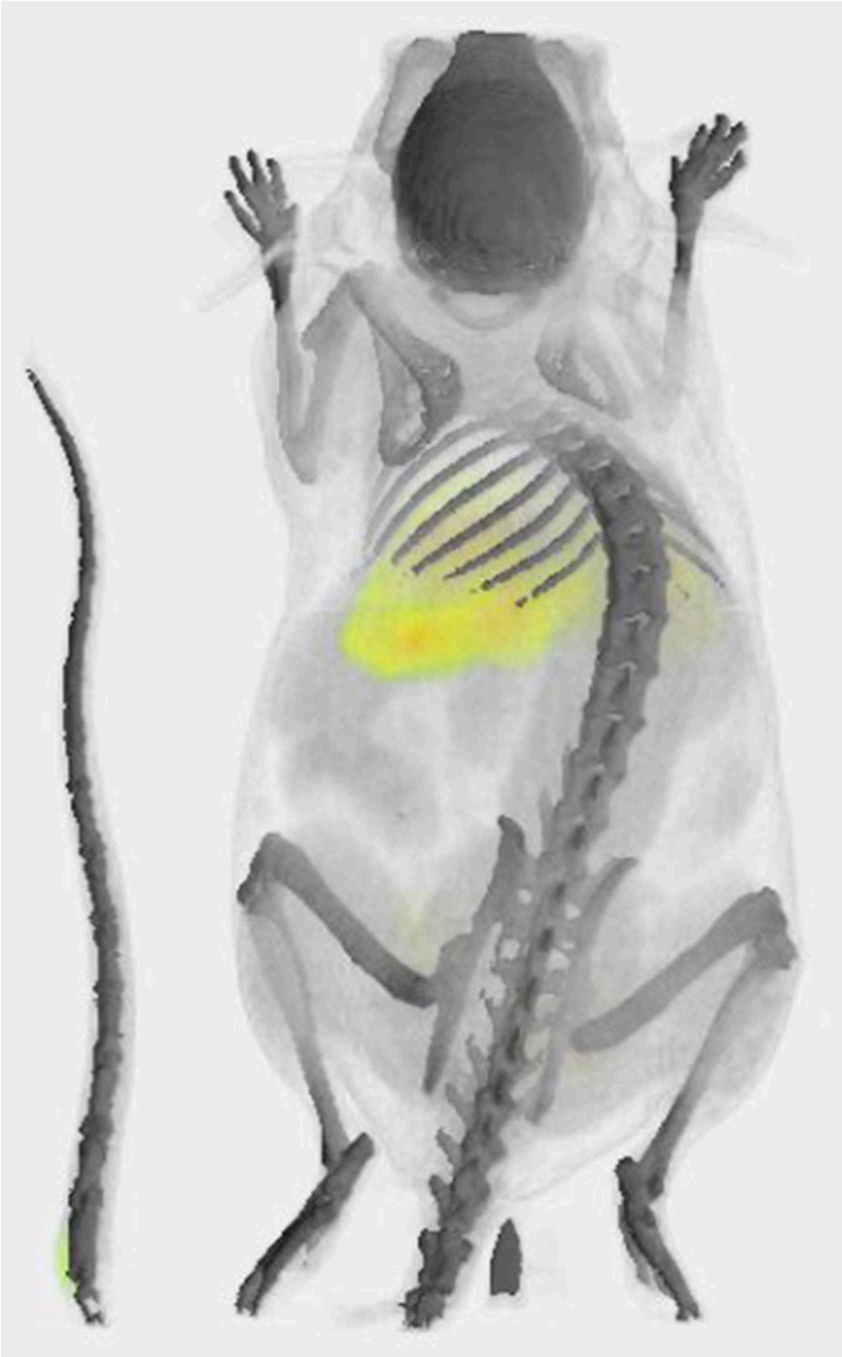

Supplementary Video 1: *In vivo* imaging of LinTT1-Tyr-^{124}I-PS. Video of the 3D reconstruction from PET-CT images after 48 hours of LinTT1-Tyr-^{124}I-PS injection in 4T1 tumor bearing mouse. The breast tumor is located in the lower orange area. A Gauss Filter of 1.5mm^3 was applied to the PET image in order to increase the signal to noise ratio for 3D visualization proposes.

See Supplementary Video 1
